# Cellular and Mitochondrial Effects of Alcohol Consumption

**DOI:** 10.3390/ijerph7124281

**Published:** 2010-12-21

**Authors:** Salvador Manzo-Avalos, Alfredo Saavedra-Molina

**Affiliations:** Instituto de Investigaciones Quimico-Biologicas, Universidad Michoacana de San Nicolas de Hidalgo, Edificio B-3. C.U., 58030 Morelia, Michoacan, Mexico; E-Mail: smanzo@umich.mx

**Keywords:** alcohol consumption, oxidative stress, mitochondria, antioxidants

## Abstract

Alcohol dependence is correlated with a wide spectrum of medical, psychological, behavioral, and social problems. Acute alcohol abuse causes damage to and functional impairment of several organs affecting protein, carbohydrate, and fat metabolism. Mitochondria participate with the conversion of acetaldehyde into acetate and the generation of increased amounts of NADH. Prenatal exposure to ethanol during fetal development induces a wide spectrum of adverse effects in offspring, such as neurologic abnormalities and pre- and post-natal growth retardation. Antioxidant effects have been described due to that alcoholic beverages contain different compounds, such as polyphenols as well as resveratrol. This review analyzes diverse topics on the alcohol consumption effects in several human organs and demonstrates the direct participation of mitochondria as potential target of compounds that can be used to prevent therapies for alcohol abusers.

## 1. Introduction

### 1.1. Alcoholism

Over the centuries, alcohol has become the most socially-accepted addictive drug worldwide [1]. Excessive alcohol use is the third leading cause of preventable death in the United States [2]. Although normative alcohol use is ubiquitous, alcohol dependence is a serious medical illness [3], experienced by ≈14% of alcohol users [4]. Alcohol dependence constitutes a substantial health and economic burden, costing an estimated $184 billion in expenditures stemming from alcohol-related chronic diseases such as heart disease [5], Alzheimer’ s disease [6], stroke [7], liver disease [8], cancer [9], chronic respiratory disease [10], diabetes mellitus [11] and bone disease [12], which may develop following chronic alcohol ingestion and contribute to the alcoholism-related high morbidity and mortality. Alcohol abuse may also trigger a cascade of acute health problems such as traffic accident-related injuries, social problem including domestic violence, loss of work-place productivity, economic burden on society, crime and public disorder [13].

Alcohol use is characterized by central nervous system (CNS) intoxication symptoms, impaired brain activity, poor motor coordination, and behavioral changes [14], largely as a result of impaired CNS activity due to alcohol’s effect on synthesis [15], release [16] and signaling [17] of neurotransmitters, including serotonin [18], glutamate [19], GABA [20], endocannabinoids [21] and their receptors. Alcohol abuse causes functional impairment of the gastrointestinal tract [22], liver [23], and pancreas [24]. It also affects protein, carbohydrate, and fat metabolism [25], and leads to insufficient immune system responses to infections [26], impairs the ability of the host to counteract hemorrhagic shock [27], augments corticosteroid release [28], and delays wound healing [29], thus contributing to higher morbidity and mortality, and prolonged recovery from trauma [30].

Alcohol intoxication results as a tolerance and physical dependence. Tolerance is defined as a reduced response to a constant amount of ethanol or an increase in the amount necessary to elicit the same effect [31]. Dependence is characterized by a withdrawal syndrome upon cessation of ethanol exposure [31]. Although significant progress had been made in the area of alcohol research during the past several decades, the pathogenesis of alcohol use and abuse is not fully understood. Understanding the mechanism that leads to tolerance and dependence may give valuable insight into alcohol addiction and ultimately result in effective therapeutic intervention to combat this disorder [31].

### 1.2. Alcohol Metabolism

The effects of alcohol on various tissues depend on its concentration in the blood over time. After oral administration, ethanol is readily absorbed by the gastrointestinal tract; absorption takes place by passive diffusion through the stomach wall (about 20%), being the remaining 80% absorbed through the duodenum and small intestine wall [32]. Elimination of absorbed ethanol occurs primarily through metabolism (95–98%), with small fractions of the administered dose being excreted unchanged in the breath (0.7%), sweat (0.1%), and urine (0.3%) [33].

**Figure 1:**
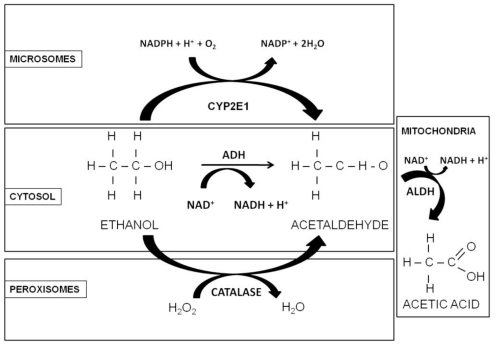
Oxidative pathways of alcohol metabolism. The enzymes alcohol dehydrogenase (ADH), cytochrome P450 2E1 (CYP2E1), and catalase all contribute to oxidative metabolism of alcohol. ADH, present in the cytosol, converts ethanol to acetaldehyde. This reaction involves an intermediate carrier of electrons, nicotinamide adenine dinucleotide (NAD^+^), which is reduced by two electrons to form NADH. Catalase, located in peroxisomes, requires hydrogen peroxide (H_2_O_2_) to oxidize alcohol. CYP2E1, present predominantly in the cell’s microsomes metabolize alcohol to acetaldehyde at elevated ethanol concentrations. Acetaldehyde is metabolized mainly by aldehyde dehydrogenase 2 (ALDH2) in the mitochondria to form acetate and NADH (Adapted from [34]).

In adult nonalcoholic individuals, most ethanol metabolism occurs in the liver, mainly via oxidation catalyzed by alcohol dehydrogenase (ADH), aldehyde dehydrogenase (ALDH), cytochrome P450 2E1 (CYP2E1), and catalase [34,35] enzymes (Figure 1). In people who consume alcohol at moderate levels (or only occasionally), the ethanol (CH_3_CH_2_OH) is oxidized to acetaldehyde (CH_3_CHO), in a reversible reaction catalyzed by class I ADH in the cytosol of hepatocytes [36]. Due to high affinity (Km = 0.05–0.1 g/L) and low capacity the enzyme becomes saturated after only few drinks. Subsequently, the acetaldehyde is then oxidized in a nonreversible reaction to acetate by the mitochondrial isoform of ALDH. Since acetaldehyde is highly toxic, it must be eliminated soon after its formation, therefore this enzyme has a very low Km and the elimination of acetaldehyde is very efficient [36]. The activated form of acetate, acetyl CoA, may be further metabolized, leading to ketone bodies, amino acids, fatty acids and steroids [35]; when it is oxidized in the Krebs cycle, CO_2_ and water are formed as the end-products of ethanol oxidation [5]. Both ADH and ALDH utilize the cofactor nicotinamide adenine dinucleotide (NAD^+^), which is reduced to NADH; as a consequence, during ethanol oxidation the ratio NADH/NAD^+^ is significantly increased, altering the cellular redox state and triggering a number of adverse effects, related to alcohol consumption [32].

In people who are chronic alcohol consumers, a second pathway, the microsomal ethanol-oxidizing system (MEOS), which functions in the smooth endoplasmic reticulum of hepatocytes, helps rid the body of toxic compounds through cytochrome P450 (CYP2E1), which, like ADH, converts alcohol to acetaldehyde [6,7]. This reaction also requires oxygen and reduced NADPH to form NADP and water. This enzyme is characterized by a low affinity (Km = 0.5–0.8 g/L) with respect to ADH [36]. As a third route of ethanol metabolism, catalase located in the cell bodies called peroxisomes, is capable of oxidizing a small amount (2%) of ethanol in the presence of a hydrogen peroxide (H_2_O_2_)-generating system [35], to form acetaldehyde, without requiring NAD as a cofactor (Figure 1) [37].

Acetaldehyde and acetate, produced from the oxidative metabolism of alcohol, contribute to cell and tissue damage in various ways. Acetaldehyde has the capacity to bind to proteins such as enzymes, microsomal proteins, and microtubules. Formation of protein adducts in hepatocytes impairs protein secretion, which has been proposed to play a role in hepatomegaly [35]. It also forms adducts with the brain signaling chemical dopamine to form salsolinol, which may contribute to alcohol dependence, and with DNA to form carcinogenic DNA adducts such as 1,*N*2-propanodeoxyguanosine [35]. On the other hand, most of the acetate resulting from alcohol metabolism escapes the liver to the blood and is eventually metabolized to CO_2_ in heart, skeletal muscle, and brain cells [35]. Acetate increases blood flow into the liver and depresses the central nervous system, as well as affects various metabolic processes [35].

Besides, mitochondria have an important role in the alcohol metabolism, and their function is affected by alcohol consumption. It has been hypothesized that upon chronic alcohol intake the brain starts using acetate rather than glucose as a source of energy [35], and the accumulated acetaldehyde exerts its toxic effects by inhibiting mitochondrial reactions and functions. In addition, there is considerable evidence that chronic alcohol exposure enhances the susceptibility of cells to undergo apoptosis, therefore is important to understand the role of mitochondria during alcohol consumption and metabolism in chronic alcohol consumption.

## 2. Effects of Alcohol on Mitochondrial Biomolecules

A mitochondrion is a membrane-enclosed organelle found in most eukaryotic cells. Mitochondria are sometimes described as “cellular power plants” because they generate the majority of the cell’s supply of adenosine triphosphate (ATP), which is used as a source of chemical energy. Its importance lies in that it contains Krebs cycle enzymes, β-oxidation, oxidative phosphorylation, and components of the electron transport chain. The number of mitochondria in a cell varies widely by organism and tissue type. The mitochondria play an important role in the alcohol metabolism via the enzyme ALDH; this enzyme catalyzes the conversion of acetaldehyde into acetate. When this enzyme reaches a saturation point, the acetaldehyde escapes into the blood stream and leads damage to biomolecules such lipids, proteins and nucleic acids which results of the toxic side effects that are associated with alcohol consumption.

There is evidence that ethanol produces alterations in the mitochondrial structure and function of several organs, including liver [38], and heart [39], both in laboratory animals and humans [40]. These changes affect the mitochondrial function decreasing respiratory rates [41] and ATP levels, and might result in increased production of reactive oxygen species (ROS) [42]. Several enzymatic systems, including the cytochrome P450 (CYP2E1), the mitochondrial respiratory chain and the cytosolic enzymes xanthine oxidase and the aldehyde oxidases have been implicated as sources of superoxide anion (O_2_ ^•−^) and H_2_O_2_ in parenchyma cells during ethanol intoxication [43]. Numerous studies shows that mitochondrial levels of ROS may be increased by chronic alcohol consumption as a consequence of increased mitochondrial CYP2E1 levels [44,45] as well as a by-product of the matrix enzyme α-ketoglutarate dehydrogenase [42]. The CYP2E1 activity increases in alcohol-treated rodents [46] and humans not only in alcohol abusers, but also in moderate alcohol consumers [47]. It has a high rate of NADPH oxidase activity, which leads to the production of large quantities of O_2_ ^•−^ and H_2_O_2_ [48]. In addition, the ethanol metabolism increases the availability of reducing equivalents (*i.e.*, NADH) to the mitochondrion, which results of the redox active semiquinone intermediates within complexes I and III to be a more “reduced” state, thereby facilitating the reduction of O_2_ to O_2_ ^•−^ [49]. Also, chronic alcohol exposure decrease the activities of all the oxidative phosphorylation complexes, except complex II [50] contributing to decreased functioning of the oxidative phosphorylation system and depressed rates of ATP synthesis [51]. As well, ethanol has been demonstrated to increase the production of ROS and reactive nitrogen species (RNS) and decrease several antioxidant mechanisms in liver [38]. This in turn might result in oxidative modification and inactivation of mitochondrial macromolecules, thereby contributing to mitochondrial dysfunction and a loss in energy conservation [38].

The mitochondrial proteome is exquisitely sensitive to modifications by ROS and RNS and thus offers an opportunity to investigate the molecular mechanisms underlying pathobiology from chronic alcohol consumption [52]. The oxidation of mitochondrial proteins is a common feature of both acute and chronic ethanol exposure [53]. Early studies by Coleman and Cunningham established a key link between the chronic alcohol-related defects in complexes I, III, IV, and V, and losses in the 13 mitochondrial encoded polypeptides and redox centers that comprises the oxidative phosphorylation system complexes [54]. Then, proteomic analysis revealed that 40 additional mitochondrial proteins had altered levels in response to chronic alcohol consumption. This includes several key energy metabolism enzymes of β-oxidation, the TCA cycle, and amino acid metabolism [55]. On the other hand, nitric oxide (NO) production is increased in response to chronic alcohol via induction of inducible nitric oxide synthase (iNOS) [56]. NO and its metabolite peroxynitrite (ONOO^−^) have been implicated as key mediators of mitochondrial dysfunction [57]. This ONOO^−^ can directly or indirectly participate in reactions leading to inactivation of mitochondrial proteins via post-translational modifications [52,58]. Also, the identification of proteins with oxidized and/or modified thiol groups are critical for elucidating the mitochondrial defects that contribute to alcoholic liver disease [52]. A number of reversible and irreversible modifications to cysteine residues are known to occur upon interaction of free sulfhydryl groups (-SH) with ROS, RNS; and reactive lipids [52]. As a consequence of oxidative modification of thiols others have shown an alcohol-dependent loss of function of the mitochondrial low Km ALDH [55]. Additionally, Moon *et al.* have demonstrated alcohol-dependent inactivation of ALDH and several β-oxidation enzymes via oxidation and nitrosation of thiols [59]. These findings are consistent with the concept that modification of protein thiols may contribute to alcoholic steatosis and mitochondrial dysfunction through inactivation of proteins critical to the energy conservation pathways in liver [52].

On the other hand, lipid peroxidation has been linked to the impairment of mitochondrial oxidative phosphorylation and the appearance of megamitochondria [60]. In patients with alcoholic liver disease (ALD) the serum markers of lipid peroxidation, such as conjugated dienes, malondialdehyde (MDA), 4-hydroxynonenal and F2-isoprostanes are increased [61]. These compounds can form adducts with proteins in the areas of fat liver infiltration, focal necrosis and fibrosis [62]. The levels of hydroxyl radicals, which exert their cytotoxic effects by causing peroxidation of membrane phospholipids, are also increased, increasing membrane permeability plus impairing membrane function [63], leading the collapse of the mitochondrial membrane potential and the onset of mitochondrial permeability transition (MPT) [53]. Other studies showed that lipoperoxidation increased the sensitivity of the electron transport chain to inhibition by oxidative stress except at the level of complex II [64]. There is evidence that oxidative stress affects the mitochondrial DNA (mtDNA).

In hepatocytes from male Wistar rats, there is a positive correlation between hepatic ATP content and the number of single-stranded DNA (ss-DNA)-positive cells. A mitochondrial function, at least in part, ATP synthesis was depressed before the damage of mtDNA by chronic ethanol consumption [65]. Mansouri *et al.* [66] found in the liver tissue and white blood cells from patients with ALD a significantly decreased mtDNA copy number and an increased level of mtDNA deletion, similar to the data obtained by Tsuchishima *et al.* [67] who also found an acquired mutation of mtDNA, at least in the encoding ATPase region, that may be reversed by stopping drinking. In addition, von Wurmb-Schwark *et al.* [68] investigated mitochondrial mutagenesis in patients with a chronic and moderate alcoholic disease, and found a relative amount of 4,977 bp deleted in mtDNA in alcoholics compared to controls.

Bailey [38] showed that there is a decrease in several antioxidant mechanisms in liver caused by increased ROS and RNA levels during chronic alcohol exposure. Early studies have shown that a decrease in the liver content or reduced GSH is a common feature in ethanol-fed animals as well as in patients with alcoholism [43]. Chronic alcohol intake lowers the mitochondrial GSH (mtGSH) [69], which makes these organelles more susceptible to oxidative damage, and precedes the development of mitochondrial dysfunctions, such as lipid peroxidation [69], and the impairment of ATP synthesis [70]. Several investigations using the enteral alcohol model [71] have shown a marked decline in enzymatic activity and immunoreactive protein concentrations of liver Cu, Zn superoxide dismutase (SOD), catalase and GSH peroxidase, suggesting that ethanol might interfere at the post-transcriptional level with the synthesis of antioxidant enzymes or might stimulate their intracellular degradation [43].

**Figure 2:**
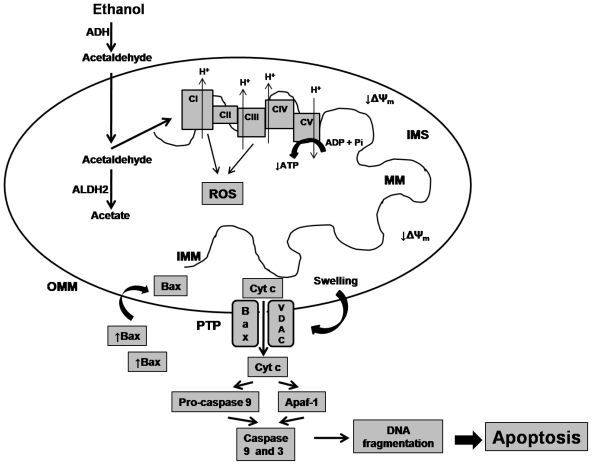
Ethanol effects on mitochondrial function. Alcohol is metabolized to acetaldehyde by the cytosolic enzyme alcohol dehydrogenase (ADH). Mitochondrial aldehyde dehydrogenase 2 (ALDH2) converts acetaldehyde to acetate. When this enzyme is malfunctioning, acetaldehyde increases and damages the electron transport complexes (CI-CIV) leading over production of reactive oxygen species (ROS), affecting electron transport chain (ETC) and oxidative phosphorylation disturbing ATP synthesis. Also, oxidative stress affects the permeability of the outer/inner mitochondrial membranes (OMM/IMM) promoting opening of the permeability transition pore (PTP) favoring the translocation of the pro-apoptotic factor bax, which forms a complex with voltage-dependent anion channel (VDAV). When the mitochondrial permeability transition is extensive, promotes the mitochondrial swelling and permits the cytochrome *c* release (Cyt *c*), caspase activation and DNA fragmentation, leading the programmed cell death or apoptosis. MM, mitochondrial matrix; ΔΨm, mitochondrial membrane potential. IMS, intramitochondrial space; Apaf-1, Apoptotic protease activating factor-1; ATP, adenosine triphosphate; ADP, adenosine diphosphate.

The damage accumulated in biomolecules triggered by acetaldehyde exerts its toxic effects by inhibiting the mitochondrial reactions and functions (Figure 2). This compound may injure the electron transport chain (ETC) function, leading to production of ROS, which can oxidize the subunits of ETC complexes, leading injury over electron transport and oxidative phosphorylation [72,73], therefore decreasing the ATP levels. In addition, the ROS may lead oxidative stress over lipids causing lipid peroxidation, which affects the permeability of the outer and/or inner mitochondrial membranes. These allows opening of the mitochondrial permeability transition pore (MPTP) and lead to mitochondrial permeability transition (MPT), favoring the translocation to the mitochondria of the pro-apoptotic factor Bax that forms a complex with a voltage-dependent anion channel (VDAC). Extensive MPT leads to mitochondria swelling as a result of the influx of ions and water, and permits the cytochrome *c* release [74], leading to caspases activation [75] and DNA fragmentation, which are key events for induction of programmed cell death or apoptosis [74].

## 3. Alcohol Effects on the Heart

Chronic alcohol abuse has been established as a major cause of cardiomyopathy in humans [76]. The heart becomes enlarged and has flaccid muscle tone, presents scattered areas of muscle degeneration, fibrosis, intracellular edema, lymphocytic infiltration and numerous fat droplets are observed in muscle-cell cytoplasm. These changes may lead the loss of cells by either necrosis or apoptosis as a plausible mechanism for decrease in contractile function [77]. In addition, ethanol causes changes in the spatial reorganization of mitochondria: intermitochondrial contacts disappear, and the mitochondrial population regroups into separate clusters uniformly distributed over the space of a muscle cell. Subsequently, megamitochondria and septate mitochondria appear. These changes may be considered a sign of impairments of myocardial functioning. Data of animal experiments show a decrease in the rates of respiration and oxidative phosphorylation [78]. In baboons the cytochrome *c* oxidase concentration and activity decreased two-fold, while in rats causes a decrease of oxidative phosphorylation efficiency and weakening of the factor F_1_ connection with mitochondrial membrane [79].

Alcoholic beverages contain more than 200 compounds with different antioxidant activities to polyphenols, including quercetin, catechin, tannic acids [80], and resveratrol, among others [81]. Resveratrol is a polyphenolic phytoalexin (trans-3,4′,5-trihydroxystilbene) present in purple grape juice, peanuts, and red wine [35,82] and has ability to prevent or slow the progression of a variety of pathologies [83]. It also possesses many health benefits that include cardioprotection [84]. It may reduce the incidence of coronary heart disease by its antioxidant [85] and anti-inflammatory [86] activities, preconditioning against ischemic injury [87], reduced ischemia-reperfusion injury and infarction [88], attenuated hypertrophic response [89], enhanced peri-infarct neovascularization [90], and antiarrhytmic efficacy [91], inhibit cardiac fibroblast proliferation and differentiation in vitro [92].

Resveratrol can retard progression of atherosclerosis. In apolipoprotein E-deficient mice, resveratrol reduces the susceptibility of LDL to oxidation and aggregation [93], while in vascular smooth muscle cells inhibited the platelet-derived growth factor beta receptor (PDGF) that is crucial on the development of atherosclerosis [94]. In addition, it interferes with angiotensin II and epidermal growth factor signaling in vascular smooth muscle cells, which may be a long-term mechanism for inhibition of atherosclerosis [95]. In patients with coronary artery disease resveratrol decreased arterial stiffness [96]. The supplementation of this compound controls the high cholesterol diet-induced myocardial complications such as myocardial and aortic damage in mice [97] and increases infarct size in rats [98], by regulating signal transduction pathways that leads to angiogenesis and cardioprotection in the hypercholesterolemic myocardium [99]. The enhanced neovascularization observed in the infarcted rat myocardium [100] may be due to its ability to modulate certain signal pathways of cell proliferation and survival [83].

## 4. Alcohol Effects on the Stomach

Alcohol is absorbed rapidly through the bloodstream from the stomach and intestinal tract. High concentrations of ethanol induce vascular endothelium injury of the gastric mucosa, which became edematous, and congestive, present point and scattered bleeding lesions, focal hemorrhage, necrosis, and giant and deep ulcers were visible [101]. Principal cells and parietal cells become swollen and diminished [101]. These cells are rich in mitochondria [102], which provide energy by oxidative phosphorylation, which is critical for maintaining the proper morphology and function of the gastric mucosa. The mitochondrion is an easily injured organelle, and mtDNA is the major target of ethanol-associated intracellular oxidative stress associated [103]. There are evidences that during the expression of mtDNA, the subunits 6 and 8 mRNA of ATPase decreased in ethanol-induced acute injury [101], and the lack of ATP may lead to metabolic acidosis, cellular edema, intracellular calcium overload, and further damage to gastric mucosa cells [104].

Alcohol exposure affects the mitochondrial structure which became swollen and disaggregated, and the cristae were dissolved and disappeared [101], giving rise to megamitochondria [105], which have oxygen consumption, ATP synthesis, and ROS-formation rates lower than those of controls. Therefore, it was proposed that enlargement of the mitochondria is an adaptive process by which cells attempt to decrease the intracellular amount of ROS when they are subjected to oxidative stress [106]. Gastric mucosa is rich in protein sulfhydryl groups, which may be the target of ROS. Oxidized protein sulfhydryl groups lead to protein denaturation or enzyme inactivation and receptor damage or modification of the cell membrane, thus contributing to mucosal injury [107].

## 5. Alcohol Effects on the Liver

Alcoholic liver disease is damage to the liver due to alcohol abuse and usually occurs after years of excessive drinking. Changes in the liver include steatosis, steatohepatitits to fibrosis and cirrhosis [108,109]. Moreover, chronic alcohol consumption is an established risk factor for the development of hepatocellular carcinoma in patients with liver cirrhosis [110].

In early stages of the ALD, the alcoholic steatosis is the initial pathology characterized by the accumulation of lipids in the liver. The progression to alcoholic steatohepatitis represents the key step in the development of ALD, where hepatic stellate cells are activated and recognized as fibrogenic cells and lead to deposition of collagen [111]. Also, activated Kupffer cells secrete pro-inflammatory cytokines, linking apoptosis in the liver to inflammation [112]. When ALD is established, an accumulation of reducing equivalents in the cytosol and the rates of fatty acids biosynthesis and subsequent esterification into triglycerols are increased [113]. It is also possible to observe massive hepatocyte apoptosis, which induces progressive fibrosis, and could result in liver failure, cirrhosis, and hepatocellular carcinoma [111].

Liver injury mediated by alcohol involves both liver parenchymal and nonparenchymal cells, including resident and recruited immune cells that contribute to liver damage and inflammation [114]. Biopsies from patients with ALD showed partial villous atrophy, increase in lamina propia infiltrate, and intraepithelial lymphocytes. Ultrastructural evaluation revealed changes such as widened intercellular junction, distorted microvilli, increased rough endoplasmic reticulum, and increased and dilated mitochondria [115]. Chronic alcohol administration favors the formation of megamitochondria, due to increasing mitochondrial membrane permeability and decreasing mitochondrial membrane potential [116] and diminished activity of mitochondrial respiratory chain complexes [117].

Mitochondrial and cellular oxidative stress in chronic alcoholism appears to be the major cause of augmented mitochondrial production of superoxide anion (O_2_ ^•−^) at complexes I and III, and consequently the production of H_2_O_2_ and other ROS, triggered by NADH overproduction. Mitochondrial oxidative stress renders hepatocytes susceptible to ethanol- or acetaldehyde-induced mitochondrial membrane permeability transition (MMPT), apoptosis in chronic alcoholism and biliary cirrhosis [118]. Through phosphorylation/dephosphorylation of Bcl-2 proteins, chronic ethanol may control the sensitivity of mitochondria toward a variety of membrane permeabilization-inducing factors [119].

## 6. Alcohol Effects on the Nervous System

For a long time the effect of alcohol was thought to be a generalized depression of neural activity causing global impairment of cognitive, psychological, and behavioral domains [120]. Recently, it has been shown that ethanol can alter mentation in a variety of ways affecting many neurotransmitter systems [121]. Early symptoms of acute intoxication are euphoria and disinhibition, which progress to stupor and respiratory depression [122]. Abrupt abstinence after prolonged or binge drinking can result in tremors, hallucinations (visual, auditory, or tactile), seizures, or delirium tremens, with severely constricted attentiveness, fluctuating levels of alertness, agitation, and autonomic instability [123]. It is possible, moreover, that repeated binges and withdrawals cause permanent neuronal damage contributing to more lasting neurological disorders, including dementia [124].

Animal experiments have demonstrated that bouts of binge drinking can produce necrotic neurodegeneration in brain areas most closely associated with the hippocampus [125]. Acute ethanol administration produces dose-dependent impairments in spatial learning, and decreases the spatial specificity of hippocampus place cells [126]. Additionally, adult neurogenesis within the hippocampus’ dentate gyrus is selectively impaired in a rat model of alcoholism. This impaired neurogenesis may be a mechanism that mediates the cognitive deficits observed in alcoholism, thus agree with the hypothesis that alcohol interferes with learning processes and memory recall [127].

To date, the exact mechanism of action of this compound is unknown, but it has been observed that it acts on gamma amino butyric acid (GABA) receptors by enhancing the effects of GABA, producing an anxiolytic effect. It also blocks the binding of glutamate to its receptor, *N*-methyl-d-aspartate (NMDA), and reversibly reduces sodium transport in neurotransmission [128] and voltage-dependent calcium channels blocking, thus inhibiting the release of neurotransmitters [129], such as serotonin, acetylcholine, dopamine, noradrenaline, endorphin, encephalin, and neuropeptide Y [128].

Recent investigations have suggested that ethanol influences on special transmitter systems and mechanisms of formation of morphine-analogous condensation products are presented in addiction [130]. During development of alcoholism there is progressive accumulation of acetaldehyde and a parallel increase of dopamine concentration in blood creating conditions for the condensation of acetaldehyde with dopamine [131] producing tetrahydropapaveroline [132], which is an intermediate in the biosynthesis of morphine [133]. Also, biogenic amines may react with acetaldehyde to form isoquinoline or carboline compounds, which may enter neural stores and displace the natural neurotransmitter, thus they can act as false neurotransmitters [132]. These results suggest that these compounds may be responsible for development of alcohol addiction. In addition, as products of alcohol metabolism also generates ROS and nitric oxide (NO) via induction of NADPH/xanthine oxidase and nitric oxide synthase (NOS) in human neurons contributing to oxidative and nitrosative stress [134].

Brain mitochondria appear to be the principal targets of the oxidative stress generated by ethanol intoxication and withdrawal. This stress causes extensive degradation and depletion of brain mtDNA in mice [135] and decreases cytochrome *c* oxidase (COX) activity in a variety of neurodegenerative illnesses, such Parkinson disease and Alzheimer disease (AD). Upon removal of chronic ethanol, excessive glutamate-induced neuronal excitation, increases intracellular concentrations of Ca^2+^ and ROS, factors that provoke PTP opening, allowing for non-selective passage of solutes and water, leading mitochondrial swelling and possible rupture and decreased efficiency of mitochondrial respiration [136,137]. In AD, epidemiological studies have indicated that alcohol consumption plays a role in the development of the disease, due to enhances beta-amyloid (Abeta)-induced neuronal cell death by increasing ROS and mitochondrial dysfunction [138].

## 7. Alcohol and Prenatal Effects

Due to its soluble nature, alcohol does not bind to any tissue nor is it bound to plasma proteins, but can cross the blood brain barrier and placenta [139]. Prenatal exposure to ethanol during development induces a wide spectrum of adverse effects in offspring; the most extreme of which is fetal alcohol syndrome (FAS), a condition characterized by microcephaly, neurologic abnormalities, facial dysmorphology, and pre- and post-natal growth retardation [139]. Neuropathologic abnormalities in FAS include neuronal-glial heterotopias, cerebellar dysplasia, and agenesis of the corpus callosum, hydrocephalus, and microcephaly [140]. These lesions are indicative of aberrant migration, decreased proliferation, and the death of neuronal cells. Pregnant women are well advised to abstain from drinking ethanol, due to that serotonin (a trophic factor for brain development) levels are significantly decreased in a ethanol-exposed fetus [141] and reduces the number of developing serotonin (5-HT)-containing neurons by increasing apoptosis. Also, serotonin reduces several prosurvival proteins, such as Bcl-2 [142]. Hence, alcohol may affect the growth of the fetus’s forebrain through its effect on 5-HT signaling [143].

It has been postulated that neuronal alterations found in FAS could be due to some initial damage during development on astrocytes, which are more susceptible to the toxic effect of ethanol during proliferation than during differentiation. The number of mitochondria was lower and they were more elongated [144]. Electron microscopic studies on fetal rat hepatocytes illustrated a slight disruption of mitochondrial structure such as enlargement of mitochondria and dilation of cristae [145]. This disruption was accompanied by mitochondrial swelling, altered mitochondrial membrane potential, decrease in succinate dehydrogenase activity, and decrease in cellular ATP levels [145]. There are evidences that chronic ethanol intake during pregnancy in rats increased fetal liver aldehyde dehydrogenase in the mitochondrial fraction, in which the activity was 10-fold higher than in the placenta mitochondrial fraction [146].

## 8. Alcoholism Therapeutics at the Mitochondrial Level

It is important to understand the basic mechanisms of alcohol metabolism by the mitochondria, as well as the effects of alcohol on their functioning in order to develop new therapies for the treatment of alcohol addiction. Currently, among techniques used to prevent alcohol addiction-associated cellular injury, it has been employed the compound curcumin, a polyphenolic phytochemical that is extracted from the ryzomes of *Curcuma longa*, a perennial herb distributed throughout the tropical and subtropical regions of the world and commonly used in India as a spice and medical agent. Curcumin is known to protect the liver, pancreas, and nervous system from toxic effects caused by alcohol consumption [147]. Numerous studies highlighted that curcumin is a potent scavenger of a variety of ROS, such as superoxide anion, hydroxyl radicals, and nitric oxide [147]. Among of the antioxidant properties of curcumin attributed include inhibition of the oxidative damage of proteins and the peroxidation of membrane lipids in rat liver mitochondria [148], the H-atom abstraction from the phenolic OH groups present in its molecular structure [149], and chelating of metal ions such as iron [150].

The well documented cardioprotective effects of moderate alcohol consumption in animal models and in humans [149] are due to increased blood pressure and also those antioxidants properties, which can prevent oxidative stress. Resveratrol possesses diverse biochemical and physiological actions that include the ability to protect brain, kidney, and heart from ischemic injury [150]. It has estrogenic, antiplatelet, and anti-inflammatory properties [151]. The cardioprotective effects of resveratrol have been attributed to their antioxidant and anti-inflammatory properties [152,153]. This compound inhibits angiotensin II (A-II)-induced cardiomyocyte hypertrophy, because it inhibits production of ROS [154] and reduces 4-hydroxy-2-nonenal (HNE) levels in hearts from spontaneously hypertensive rats [155] maybe by increasing plasma antioxidant capacity [156] and over expression of mitochondrial superoxide dismutase, which improved respiration and normalized mass mitochondria in diabetic mice [99]. In addition, the beneficial role of resveratrol may be due also to increasing mitochondrial number as observed in obese mice [157].

Piceatannol (3,3′,4′,5-tetrahydroxystilbene, astinginin) is a resveratrol derivative with higher antioxidant capacity, found in the seeds of *Euphorbia lagascae* [157]. Piceatannol possesses an additional hydroxyl group than resveratrol (3,5,4′-trihydroxystilbene) and exerts higher radical scavenging activity which was considered to contribute to the cardioprotective and antiarrhythmic effects in ischaemic-reperfused rat heart [158,159]. Another potential compound in mitochondrial therapeutics is the structural GABA analogue citrocard (phenibut citrate) that prevents the damaging effect of alcohol, which was observed from increased indexes of oxidative phosphorylation in treated animals [78].

Ethanol-related mitochondrial dysfunction has been considered one of the major mechanisms contributing to lipid metabolism changes in the liver leading to steatosis [160]. Treatment of steatosis with IL-6 induces expression of anti-apoptotic Bcl-xL protein in primary mouse hepatocytes [161], which protects against ethanol-induced oxidative stress and mitochondrial injury in the liver [161].

Acetaldehyde is a reactive and toxic metabolite of ethanol that could affect drinking behavior and susceptibility to alcoholism. Acetaldehyde is converted into acetate by cytosolic or mitochondrial aldehyde dehydrogenase (ALDH). Mitochondrial ALDH (ALDH2) might be responsible for 60% of acetaldehyde metabolism. There is evidence that the ALDH2*2 gene encoding the inactive variant of ALDH2 protects nearly all carriers of this gene from alcoholism. Inhibition of ALDH2 has thus become a possible strategy to treat alcoholism [162].

Other agents that have received considerable attention in recent years as a potential therapeutic against alcohol-induced organs injury are betaine and *S*-adenosyl-l-methionine, which have beneficial effects on mitochondrial functions. Betaine, also known as trimethylglycine, is a methyl donor, which can replace folate or *S*-ademethionine in the human body [163], where it participates in the methionine recycling, and phosphatidylcholine synthesis [164]. Many studies have indicated that betaine can prevent the alcohol-induced injury and improve the cellular function [165] through the inhibition of inflammatory factor, the decrease of lipid peroxidation, and prevents apoptosis [166]. The other compound, *S*-adenosyl-l-methionine (SAM), present in all living cells, is synthesized from methionine and ATP [167] by the enzyme methionine adenosyltransferase (MAT) [168]. Its biochemical functions are: (1) a donor of methyl groups; (2) a sulfur-containing metabolite for the transsulfuration pathway that leads to the synthesis of cysteine and glutathione; and (3) a precursor molecule for the aminopropylation pathway that provokes the synthesis of polyamines [168]. SAM plays an important role in regulating mitochondrial function [169], due to the fact that SAM prevents alcohol-dependent mitochondrial dysfunction via the preservation of mitochondrial respiration, attenuation of mitochondrial O_2_^•−^ production, and maintenance of the integrity of the mtDNA [170].

## 9. Concluding Remarks

The metabolism of ethanol is closely linked with stimulation of reactive oxygen species generation and oxidative stress. The ability of alcohol to promote oxidative stress and the role of ROS in alcohol-induced tissue injury clearly are important areas of research in the alcohol field, particularly because such information may be of major therapeutic significance in the development of more effective and selective new medications capable of blocking the actions of ROS and consequently the toxic effects of alcohol. This knowledge will clearly advance the design and testing of novel mitochondria-specific therapeutics on the treatment of diseases in alcoholic patients. The identification of possible biomarkers of susceptibility will represent the main goal in the near future and will contribute to the implementation of adequate prevention strategies, to the development of effective diagnostic test strategies, to detect higher risk drinking behavior and early indicators of tissue damage.

## References

[1] Guo R Ren J Alcohol and acetaldehyde in public health: From marvel to menace Int. J. Environ. Res. Public Health 2010 7 1285 1301 20617031 10.3390/ijerph7041285 PMC2872347

[2] Mokdad AH Marks JS Stroup DF Gerberding JL Actual causes of death in the United States JAMA 2000 291 1238 1245 15010446 10.1001/jama.291.10.1238

[3] Diagnostic and Statistical Manual of Mental Disorders4th ed American Psychiatric Association Washington, DC, USA 1994

[4] Grant BF Stinson FS Harford TC Age at onset of alcohol use and DSM-IV alcohol abuse and dependence: A 12-year follow-up J. Substain. Abuse 2001 13 493 504 10.1016/s0899-3289(01)00096-7 11775078

[5] George A Figueredo VM Alcohol and arrhythmias: A comprehensive review J. Cardiov. Med. (Hagerstown) 2010 11 221 228 10.2459/JCM.0b013e328334b42d 19923999

[6] Marinho V Laks J Engelhardt E Conn D Alcohol abuse in an elderly woman taking donepezil for Alzheimer disease J. Clin. Psychopharmacol 2006 26 683 685 17110839 10.1097/01.jcp.0000246213.41647.9f

[7] Ohkubo T Metoki H Imai Y Alcohol intake, circadian blood pressure variation, and stroke Hypertension 2009 53 4 5 19029484 10.1161/HYPERTENSIONAHA.108.123018

[8] Cederbaum AI Lu Y Wu D Role of oxidative stress in alcohol-induced liver injury Arch. Toxicol 2009 83 519 548 19448996 10.1007/s00204-009-0432-0

[9] Seitz HK Becker P Alcohol metabolism and cancer risk Alcohol Res. Health 2007 30 38 47 17718399 PMC3860434

[10] Morris MJ Alcohol breath testing in patients with respiratory disease Thorax 1990 45 717 721 2247860 10.1136/thx.45.10.717 PMC462712

[11] Baliunas DO Taylor BJ Irving H Roerecke M Patra J Mohapatra S Rehm J Alcohol as a risk factor for type 2 diabetes: A systematic review and meta-analysis Diabetes Care 2009 32 2123 2132 19875607 10.2337/dc09-0227 PMC2768203

[12] Chen Y Cui L Liao J huang L Effects of alcohol on bone metabolism and biomechanical property of mice Sheng Wu Yi Xue Gong Cheng Xue Za Zhi 2009 26 780 782 19813609

[13] Rehm J Mathers C Popova S Thavorncharoensap M Teerawattananon Y Patra J Global burden of disease and injury and economic cost attributable to alcohol use and alcohol-use disorders Lancet 2009 373 2223 2233 19560604 10.1016/S0140-6736(09)60746-7

[14] Gohlke JM Griffith WC Faustman EM Computational models of ethanol-induced neurodevelopmental toxicity across species: Implications for risk assessment Birth Defects Res. B. Dev. Reprod. Toxicol 2008 83 1 11 18161053 10.1002/bdrb.20137

[15] Pietrzykowski AZ Friesen RM Martin GE Puig SI Nowak CL Wynne PM Siegelmann HT Treistman SN Posttranscriptional regulation of BK channel splice variant stability by miR-9 underlies neuroadaptation to alcohol Neuron 2008 59 274 287 18667155 10.1016/j.neuron.2008.05.032 PMC2714263

[16] Roberto M Treistman SN Pietrzykowski AZ Weiner J Galindo R Mameli M Valenzuela F Zhu PJ Lovinger D Zhang TA Hendricson AH Morrisett R Siggins GR Actions of acute and chronic ethanol on presynaptic terminals Alcohol Clin. Exp. Res 2006 30 222 232 16441271 10.1111/j.1530-0277.2006.00030.x PMC4115792

[17] Wilkie MB Besheer J Kelley SP Kumar S O’Buckley TK Morrow AL Hodge CW Acute ethanol administration rapidly increases phosphorylation of conventional protein kinase C in specific mammalian brain regions *in vivo* Alcohol Clin. Exp. Res 2007 31 1259 1267 17511744 10.1111/j.1530-0277.2007.00423.x PMC2861774

[18] Le Marquand D Pihl RO Benkelfat C Serotonin and alcohol intake, abuse, and dependence: Clinical evidence Biol. Psychiatry 1994 36 326 337 7993959 10.1016/0006-3223(94)90630-0

[19] Prosser RA Mangrum CA Glass JD Acute ethanol modulates glutamatergic and serotonergic phase shifts of the mouse circadian clock in vitro Neuroscience 2008 152 837 848 18313227 10.1016/j.neuroscience.2007.12.049 PMC2377014

[20] Wallner M Hanchar HJ Olsen RW Low-dose alcohol actions on alpha4beta3delta GABAA receptors are reversed by the behavioral alcohol antagonist Ro15-4513 Proc. Natl. Acad. Sci. USA 2006 103 8540 8545 16698930 10.1073/pnas.0600194103 PMC1482527

[21] Perra S Pillolla G Luchicchi A Pistis M Alcohol inhibits spontaneous activity of basolateral amigdala proyection neurons in the rat: Involvement of the endocannabinoid system Alcohol Clin. Exp. Res 2008 32 443 449 18215217 10.1111/j.1530-0277.2007.00588.x

[22] Fisher SJ Lee IJ Swaan PW Eddington ND Evaluation of the effect of ethanol’s toxic metabolite acetaldehyde on the gastrointestinal oligopeptide transporter, PEPT1: *In vitro* and *in vivo* studies Alcohol Clin. Exp. Res 2008 32 162 170 18028524 10.1111/j.1530-0277.2007.00551.x

[23] Karinch AM Martin JH Vary TC Acute and chronic ethanol consumption differentially impact pathways limiting hepatic protein synthesis Am. J. Physiol. Endocrinol. Metab 2008 295 E3 E9 18334613 10.1152/ajpendo.00026.2008 PMC2493597

[24] Yang AL Vadhavkar S Singh G Omary MB Epidemiology of alcohol-related liver and pancreatic disease in the United States Arch. Intern. Med 2008 168 649 656 18362258 10.1001/archinte.168.6.649

[25] Ting JW Lautt WW The effect of acute, chronic, and prenatal ethanol exposure on insulin sensitivity Pharmacol. Ther 2006 111 346 373 16310255 10.1016/j.pharmthera.2005.10.004

[26] Happel KI Rudner X Quinton LJ Movasshaghi JL Clark C Odden AR Zhang P Bagby GJ Nelson S Shellito JE Acute alcohol intoxication suppresses the pulmonary ELR-negative CXC chemokine response to lipopolysaccharide Alcohol 2007 41 325 333 17889309 10.1016/j.alcohol.2007.06.002 PMC2044567

[27] Greiffensstein P Mathis KW Stouwe CV Molina PE Alcohol binge before trauma/hemorrhage impairs integrity of host defense mechanisms during recovery Alcohol Clin. Exp. Res 2007 31 704 715 17374050 10.1111/j.1530-0277.2007.00355.x

[28] Choudhry MA Li X Chaudry IH A role for corticosterone in impaired intestinal immunity and barrier function in a rodent model of acute alcohol intoxication and burn injury J. Neuroimmune Pharmacol 2006 1 428 434 18040815 10.1007/s11481-006-9031-5

[29] Radek KA Kovacs EJ DiPietro LA Matrix proteolytic activity during wound healing: Modulation by acute ethanol exposure Alcohol Clin. Exp. Res 2007 31 1045 1052 17403061 10.1111/j.1530-0277.2007.00386.x

[30] Dolganiuc A Szabo G *In vitro* and *in vivo* models of acute alcohol exposure World J. Gastroenterol 2009 15 1168 1177 19291816 10.3748/wjg.15.1168 PMC2658858

[31] Werner DF Swihart AR Ferguson C Lariviere WR Harrison NL Homanics GE Alcohol-induced tolerance and physical dependence in mice with ethanol insensitive α1 GABA_A_ receptors Alcohol Clin. Exp. Res 2009 33 289 299 19032579 10.1111/j.1530-0277.2008.00832.x PMC2786059

[32] Norberg A Jones AW Hahn RG Gabrielsson JL Role of variability in explaining ethanol pharmacokinetics: Research and forensic applications Clin. Pharmacokinet 2003 42 1 31 12489977 10.2165/00003088-200342010-00001

[33] Holford NHG Clinical pharmacokinetics of ethanol Clin. Pharmacokinet 1987 13 273 292 3319346 10.2165/00003088-198713050-00001

[34] Zakhari S Overview: How is alcohol metabolized by the body? Alcohol Res. Health 2006 29 245 254 17718403 PMC6527027

[35] Agarwal DP Genetic polymorphisms of alcohol metabolizing enzymes Pathol. Biol 2001 49 703 709 11762132 10.1016/s0369-8114(01)00242-5

[36] Gemma S Vichi S Testai E Individual susceptibility and alcohol effects: Biochemical and genetic aspects Ann. Ist. Super. Sanita 2006 42 8 16 16801720

[37] Weiner H Subcellular localization of acetaldehyde oxidation on liver Ann. NY Acad. Sci 1987 492 25 34 3474929 10.1111/j.1749-6632.1987.tb48650.x

[38] Klein H Harmjanz D Effect of ethanol infusion on the ultrastructure of human myocardium Postgrad. Med. J 1975 51 325 329 1215245 10.1136/pgmj.51.595.325 PMC2495975

[39] Regan TJ Alcohol and the cardiovascular system JAMA 1990 264 377 381 2194048

[40] Pachinger O Mao J Fauvel J-M Bing RJ Fleckstein A Dhalla NS Mitochondrial function and excitation-contraction coupling in the development of alcoholic cardiomypathy Recent Advances in Studies on Cardiac Structure and Metabolism University Park Press Baltimore, MD, USA 1975 5 423 429 1237922

[41] Albano E Sherman VR Watson RR Free radicals and alcohol-induced liver injury Ethanol and the Liver Taylor and Francis London, UK 2002 153 190

[42] Robin MA Sauvage I Grandperret T Descatoire V Pessayre D Fromenty B Ethanol increases mitochondrial cytochrome P450 2E1 in mouse liver and rat hepatocytes FEBS Lett 2005 579 6895 6902 16337197 10.1016/j.febslet.2005.11.029

[43] Cederbaum AI Microsomal generation of reactive oxygen species and their possible role in alcohol hepatoxicity Alcohol Alcohol 1991 Suppl 1 291 296 1669007

[44] Adam-Vizi V Production of reactive oxygen species in brain mitochondria: Contribution by electron transport chain and non-electron transport chain sources Antioxid. Redox Signal 2005 7 1140 1149 16115017 10.1089/ars.2005.7.1140

[45] Ronis MJJ Lindros KO Ingelman-Sundberg M Ioannides C The CYP2E family Cytochromes P450: Metabolic and Toxicological Aspects CRC Press Boca Raton, FL, USA 1996 211 239

[46] Liangpunsakul S Kolwankar D Pinto A Gorski CJ Hall SD Chalasani N Activity of CYP2E1 and CYP3A enzymes in adults with moderate alcohol consumption: A comparison with nonalcoholics Hepatology 2005 41 1144 1150 15841467 10.1002/hep.20673

[47] Albano E Alcohol, oxidative stress and free radical damage Proc. Nutr. Soc 2006 65 278 290 16923312 10.1079/pns2006496

[48] Bailey SM Pietsch EC Cunningham CC Ethanol stimulates the production of reactive oxygen species at mitochondrial complexes I and III Free Radic. Biol. Med 1999 27 891 900 10515594 10.1016/s0891-5849(99)00138-0

[49] Cunningham CC Coleman WB Spach PI The effects of chronic ethanol consumption on hepatic mitochondrial energy metabolism Alcohol Alcohol 1990 25 127 136 2142884 10.1093/oxfordjournals.alcalc.a044987

[50] Bailey SM Cunningham CC Effect of dietary fat on chronic ethanol-induced oxidative stress in hepatocytes Alcohol Clin. Exp. Res 1999 23 1210 1218 10443988

[51] Bailey SM A review of the role of reactive oxygen and nitrogen species in alcohol-induced mitochondrial energy metabolism Free Radic. Res 2003 37 585 596 12868485 10.1080/1071576031000091711

[52] Mantena SK King AL Andringa KK Landar A Darley-Usmar V Bailey SM Novel interactions of mitochondria and reactive oxygen/nitrogen species in alcohol mediated liver disease World J. Gastroenterol 2007 13 4967 4973 17854139 10.3748/wjg.v13.i37.4967 PMC4434620

[53] Hoek JB Cahill A Pastorino JG Alcohol and mitochondria: A dysfunctional relationship Gastroenterology 2002 122 2049 2063 12055609 10.1053/gast.2002.33613 PMC1868435

[54] Coleman WB Cunningham CC Effect of chronic ethanol consumption on hepatic mitochondrial transcription and translation Biochim. Biophys. Acta 1991 1058 178 186 1710928 10.1016/s0005-2728(05)80235-x

[55] Venkatraman A Landar A Davis AJ Chamlee L Sanderson T Kim H Page G Pompilius M Ballinger S Darley-Usmar V Bailey SM Modification of the mitochondrial proteome in response to the stress of ethanol-dependent hepatotoxicity J. Biol. Chem 2004 279 22092 22101 15033988 10.1074/jbc.M402245200

[56] Bailey SM Robinson G Pinner A Chamlee L Ulasova E Pompilius M Page GP Chhieng D Jhala N Landar A Kharbanda KK Ballinger S Darley-Usmar V S-adenosylmethionine prevents chronic alcohol-induced mitochondrial dysfunction in the rat liver Am. J. Physiol. Gastrointest. Liver Physiol 2006 291 G857 G867 16825707 10.1152/ajpgi.00044.2006

[57] Radi R Cassina A Hodara R Quijano C Castro L Peroxynitrite reactions and formation in mitocondria Free Radic. Biol. Med 2002 33 1451 1464 12446202 10.1016/s0891-5849(02)01111-5

[58] Brookes PS Kraus DW Shiva S Doeller JE Barone MC Patel RP Lancaster JR Jr Darley-Usmar V Control of mitochondrial respiration by NO., effects of low oxygen and respiratory state J. Biol. Chem 2003 278 31603 31609 12788936 10.1074/jbc.M211784200

[59] Moon KH Hood BL Kim BJ Hardwick JP Conrads TP Veenstra TD Song BJ Inactivation of oxidized and S-nitrosylated mitochondrial proteins in alcoholic fatty liver of rats Hepatology 2006 44 1218 1230 17058263 10.1002/hep.21372

[60] Matsuhashi T Karbowski M Liu X Usukura J Wozniak M Wakabayashi T Complete suppression of ethanol-induced formation of megamitochondria by 4-hydroxy-2,2,6,6,-tetramethyl-piperidine-1-oxyl (4-OH-TEMPO) Free Radic. Biol. Med 1998 24 139 147 9436623 10.1016/s0891-5849(97)00210-4

[61] Meager EA Barry OP Burke A Lucey MR Lawson JA Rokach J FitzGerald GA Alcohol-induced generation of lipid peroxidation products in humans J. Clin. Invest 1999 104 805 813 10491416 10.1172/JCI5584 PMC408429

[62] Niemela O Parkkila S Yla-Herttuala S Halsted C Witztum JL Lanca A Israel Y Covalent protein adducts in the liver as a result of ethanol metabolism and lipid peroxidation Lab. Invest 1994 70 537 546 8176892

[63] Slater TF Free-radical mechanisms in tissue injury Biochem. J 1984 222 1 15 6383353 10.1042/bj2220001 PMC1144137

[64] Cortes-Rojo C Calderon-Cortes E Clemente-Guerrero M Estrada-Villagomez M Manzo-Avalos S Mejia-Zepeda R Boldogh I Saavedra-Molina A Elucidation of the effects of lipoperoxidation on the mitochondrial electron transport chain using yeast mitochondria with manipulated fatty acid content J. Bioenerg. Biomembr 2009 41 15 28 19224349 10.1007/s10863-009-9200-3 PMC2922399

[65] Fukumura A Tsutsumi M Tsuchishima M Takase S Correlation between adenosine triphosphate content and apoptosis in liver of rats treated with alcohol Alcohol Clin. Exp. Res 2003 27 12S 15S 12960500 10.1097/01.ALC.0000078609.36825.48

[66] Mansouri A Fromenty B Berson A Robin MA Grimbert S Beaugrand M Erlinger S Pessayre D Multiple hepatic mitochondrial DNA deletions suggest premature oxidative aging in alcoholic patients J. Hepatol 1997 27 96 102 9252080 10.1016/s0168-8278(97)80286-3

[67] Tsuchishima M Tsutsumi M Shiroeda H Yano H Ueshima Y Shimanaka K Takase S Study of mitochondrial DNA deletion in alcoholics Alcohol Clin. Exp. Res 2000 24 12S 15S 10803772

[68] Von Wurmb-Schwark N Ringleb A Schwark T Broese T Weirich S Schlaefke D Wegener R Oehmichen M The effect of chronic alcohol consumption on mitochondrial DNA mutagenesis in human blood Mutat. Res 2008 637 73 79 17767940 10.1016/j.mrfmmm.2007.07.003

[69] Hirano T Kaplowitz N Kamimura T Tsukamoto H Fernandez-Checa JC Hepatic mitocondrial GSH depletion and progression of experimental alcoholic liver disease in rats Hepatology 1992 16 1423 1428 1446896 10.1002/hep.1840160619

[70] Fernandez-Checa JC Kaplowitz N Hepatic mitochondrial glutathione: Transport and role in disease and toxicity Toxicol. App. Pharmacol 2005 204 263 273 10.1016/j.taap.2004.10.001 15845418

[71] Rouach H Fattaccioli V Gentil M French SW Morimoto M Nordmann R Effect of chronic ethanol feeding on lipid peroxidation and protein oxidation in relation to liver pathology Hepatology 1997 25 351 355 9021946 10.1002/hep.510250216

[72] Kowaltowski AJ Castillo RF Vercesi AF Mitochondrial permeability transition and oxidative stress FEBS Lett 2001 495 12 15 11322939 10.1016/s0014-5793(01)02316-x

[73] Cortes-Rojo C Clemente-Guerrero M Saavedra-Molina A Effects of D-amino acids on lipoperoxidation in rat liver and kidney mitochondria Amino Acids 2006 32 31 37 16868653 10.1007/s00726-005-0356-9

[74] Kim JS He L Lemasters JJ Mitochondrial permeability transition: A common pathway to necrosis and apoptosis Biochem. Biophys. Res. Commun 2003 304 463 470 12729580 10.1016/s0006-291x(03)00618-1

[75] Kim WH Hong F Jaruga B Zhang ZS Fan SJ Liang TJ Gao B Hepatitis B virus X protein sensitizes primary mouse hepatocytes to ethanol- and TNF-alpha-induced apoptosis by a caspase-3-dependent mechanism Cell Mol. Immunol 2005 2 40 48 16212910

[76] Urbano-Marquez A Fernandez-Sola J Effects of alcohol on skeletal and cardiac muscle Muscle Nerve 2004 30 689 707 15490485 10.1002/mus.20168

[77] Capasso JM Li P Guideri G Malhotra A Cortese R Anversa P Myocardial mechanical, biochemical, and structural alterations induced by chronic ethanol ingestion in rats Circ. Res 1992 71 346 356 1385762 10.1161/01.res.71.2.346

[78] Perfilova VN Ostrovskii OV Verovskii VE Popova TA Lebedeva SA Dib H Effect of citrocard on functional activity of cardiomyocyte mitochondria during chronic alcohol intoxication Bull. Exp. Biol. Med 2007 143 341 343 18225758 10.1007/s10517-007-0106-y

[79] Montgomery RI Coleman WB Eble KS Cunningham CC Ethanol-elicited alterations in the oligomycin sensitivity and structural stability of the mitochondrial F0.F1 ATPase J. Biol. Chem 1987 262 13285 13289 2888757

[80] Usha R Pendurthi J Todd W Vijya M Resveratrol a polyphenolic compound found in wine inhibits tissue factor expression in vascular cells. A possible mechanism for the cardiovascular benefits associated with moderate consumption of wine Arterioscler. Thromb. Vasc. Biol 1999 19 419 426 9974427 10.1161/01.atv.19.2.419

[81] Pace-Asciak CR Hahn S Diamandis EP Soleas G Goldberg DM The red wine phenolics *trans*-resveratrol and quercetin block human platelet aggregation and eicosanoid synthesis: Implications for protection against coronary heart disease Clin. Chim. Acta 1995 235 207 219 7554275 10.1016/0009-8981(95)06045-1

[82] Bertelli AAE Giovannini L Giannessi D Migliori M Bernini W Fregoni M Bertelli A Antiplatelet activity of synthetic and natural resveratrol in red wine Int. J. Tiss. Reac 1995 17 1 3 7499059

[83] Baur JA Sinclair DA Therapeutic potential of resveratrol: The *in vivo* evidence Nat. Rev. Drug Discov 2006 5 493 506 16732220 10.1038/nrd2060

[84] Howitz KT Bitterman KJ Cohen HY Lamming DW Lavu S Wood JG Zipkin RE Chung P Kisielewski A Zhang LL Scherer B Sinclair DA Small molecule activators of sirtuins extended Saccharomyces cerevisiae lifespan Nature 2003 425 191 196 12939617 10.1038/nature01960

[85] Belguendouz L Fremont L Gozzelino MT Interaction of transresveratrol with plasma lipoproteins Biochem. Pharmacol 1998 55 811 816 9586953 10.1016/s0006-2952(97)00544-3

[86] Das S Falchi M Bertelli A Maulik N Das DK Attenuation of ischemia/reperfusion injury in rats by the anti-inflammatory action of resveratrol Arzneimittelforschung 2006 56 700 706 17225566 10.1055/s-0031-1296776

[87] Das S Alagappan VK Bagchi D Sharma HS Maulik N Das DK Coordinated induction of iNOS-VEGF-KDR-eNOS after resveratrol consumption: A potential mechanism for resveratrol preconditioning of the heart Vascul. Pharmacol 2005 42 281 289 15905131 10.1016/j.vph.2005.02.013

[88] Hattori R Otani H Maulik N Das DK Pharmacological preconditioning with resveratrol: Role of nitric oxide Am. J. Physiol 2002 282 H1988 H1995 10.1152/ajpheart.01012.2001 12003802

[89] Li HL Wang AB Huang Y Liu DP Wei C Williams GM Zhang CN Liu G Liu YQ Hao DL Hui RT Lin M Liang CC Isorhapontigenin, a new resveratrol analog, attenuates cardiac hypertrophy via blocking signaling transduction pathways Free Radic. Biol. Med 2005 38 243 257 15607907 10.1016/j.freeradbiomed.2004.10.020

[90] Kaga S Zhan L Matsumoto M Maulik N Resveratrol enhances neovascularization in the infarcted rat myocardium through the induction of thioredoxin-1, heme oxygenase-1 and vascular endothelial growth factor J. Mol. Cell Cardiol 2005 39 813 822 16198371 10.1016/j.yjmcc.2005.08.003

[91] Zhang Y Liu Y Wang T Li H Wang Z Yang B Resveratrol, a natural ingredient of grape skin: Antiarrhyhtmic efficacy and ionic mechanisms Biochem. Biophys. Res. Commun 2006 340 1192 1199 16406237 10.1016/j.bbrc.2005.12.124

[92] Wang S Wang X Yan J Xie X Fan F Zhou X Han L Chen J Resveratrol inhibits proliferation of cultured rat cardiac fibroblasts: Correlated with NO-cGMP signaling pathway Eur. J. Pharmacol 2007 567 26 35 17499237 10.1016/j.ejphar.2007.04.023

[93] Hayek T Fuhrman B Vaya J Rosenblat M Belinky P Coleman R Elis A Aviram M Reduced progression of atherosclerosis in apolipoprotein E-deficient mice following consumption of red wine, or its polyphenols quercetin or catechin, is associated with reduced susceptibility of LDL to oxidation and aggregation Arterioscler. Thromb. Vasc. Biol 1997 17 2744 2752 9409251 10.1161/01.atv.17.11.2744

[94] Rosenkranz S Knirel D Dietrich H Flesch M Erdmann E Bohm M Inhibition of the PDGF receptor by red wine flavonoids provides a molecular explanation for the “French paradox” FASEB J 2002 16 1958 1960 12397093 10.1096/fj.02-0207fje

[95] Halder UG Roos TU Kontaridis MI Neel BG Sorescu D Griedling KK Vollmar AM Dirsch VM Resveratrol inhibits angiotensin II and epidermal growth factor-mediated Akt activation: Role of Gab1 and Shp2 Mol. Pharmacol 2005 68 41 48 15849355 10.1124/mol.104.005421

[96] Karatzi KN Papamichael CM Karatzis EN Papaioannou TG Aznaouridis KA Katsichti PP Stamatelopoulos KS Zampelas A Lekakis JP Mavrikakis ME Red wine acutely induces favorable effects on wave reflections and central pressures in coronary artery diseases patients Am. J. Hypertens 2005 18 1161 1167 16182104 10.1016/j.amjhyper.2005.03.744

[97] Baur JA Pearson KJ Price NL Jamieson HA Lerin C Kalra A Prabhu VV Allard JS Lopez-Lluch G Lewis K Pistell PJ Poosala S Becker KG Boss O Gwinn D Wang M Ramaswamy S Fishbein KW Spencer RG Lakatta EG Le Couteur D Shaw RJ Navas P Puigserver P Ingram DK de Cabo R Sinclair DA Resveratrol improves health and survival of mice on a high-calorie diet Nature 2006 444 337 342 17086191 10.1038/nature05354 PMC4990206

[98] Penumathsa SV Thirunavukkarasu M Koneru S Juhasz B Zhan L Pant R Menon VP Otani H Maulik N Statin and resveratrol in combination induces cardioprotection against myocardial infarction in hypercholesterolemic rat J. Mol. Cell Cardiol 2007 42 508 516 17188708 10.1016/j.yjmcc.2006.10.018 PMC1857339

[99] Penumathsa SV Maulik N Resveratrol: A promising agent in promoting cardioprotection against coronary heart disease Can. J. Physiol. Pharmacol 2009 87 275 286 19370081 10.1139/Y09-013

[100] Wagner TM Mullally JE Fitzpatrick FA Reactive lipid species from cyclooxygenase-2 inactivate tumor suppressor LKB1/STK11: Cyclopentenone prostaglandins and 4-hydroxy-2-nonenal covalently modify and inhibits the AMP kinase kinase that modulates cellular energy homeostasis and protein translation J. Biol. Chem 2006 281 2598 2604 16311241 10.1074/jbc.M509723200

[101] Pan JS He SZ Xu HZ Zhan XJ Yang XN Xiao HM Shi HX Oxidative stress disturbs energy metabolism of mitochondria in ethanol-induced gastric mucosa injury World J. Gastroenterol 2008 14 5857 5867 18855985 10.3748/wjg.14.5857 PMC2751896

[102] Yin GY Zhang WN Shen XJ Chen Y He XF Ultrastructure and molecular biological changes of chronic gastritis, gastric cancer and gastric precancerous lesions: A comparative study World J. Gastroenterol 2003 9 851 857 12679947 10.3748/wjg.v9.i4.851 PMC4611464

[103] Hoek JB Cahill A Pastorino JG Alcohol and mitochondria: A dysfunctional relationship Gastroenterology 2002 122 2049 2063 12055609 10.1053/gast.2002.33613 PMC1868435

[104] Rong Q Utevskaya O Ramilo M Chow DC Forte JG Nucleotide metabolism by gastric glands and H(+)-k(+)-ATPase-enriched membranes Am. J. Physiol 1998 274 G103 G110 9458779 10.1152/ajpgi.1998.274.1.G103

[105] Giannessi F Giambelluca MA Grasso L Scavuzzo MC Ruffoli R Curcumin protects Leydig cells of mice from damage induced by chronic alcohol administration Med. Sci. Monit 2008 14 237 242 18971866

[106] Wakabayashi T Megamitochondria formation-physiology and pathology J. Cell Mol. Med 2002 6 497 538 12611638 10.1111/j.1582-4934.2002.tb00452.x PMC6741312

[107] Dey A Cederbaum AI Alcohol and oxidative liver injury Hepatology 2006 43 S63 S74 16447273 10.1002/hep.20957

[108] Mills SJ Harrison SH Comparison of the natural history of alcoholic and nonalcoholic fatty liver disease Curr. Gastroenterol. Reports 2010 7 32 36 10.1007/s11894-005-0063-4 15701296

[109] Stewart S Jones D Day CP Alcoholic liver disease: New insights into mechanisms and preventative strategies Trends Mol. Med 2001 7 408 413 11530336 10.1016/s1471-4914(01)02096-2

[110] Morgan TR Mandayam S Jamal MM Alcohol and hepatocellular carcinoma Gastroenterology 2004 127 S87 96 15508108 10.1053/j.gastro.2004.09.020

[111] Miranda-Mendez A Lugo-Baruqui A Armendariz-Borunda J Molecular basis and current treatment for alcoholic liver disease Int. J. Environ. Res. Public Health 2010 7 1872 1888 20622998 10.3390/ijerph7051872 PMC2898022

[112] Malhi H Gores GJ Cellular and molecular mechanisms of liver injury Gastroenterology 2008 134 1641 1654 18471544 10.1053/j.gastro.2008.03.002 PMC2553363

[113] Song BJ Moon KH Olsson NU Salem N Jr Prevention of alcoholic fatty liver and mitochondrial dysfunction in the rat by long-chain polyunsaturated fatty acids J. Hepatol 2008 49 262 273 18571270 10.1016/j.jhep.2008.04.023 PMC2532851

[114] Friedman SL Molecular regulation of hepatic fibrosis, and integrated cellular response to tissue injury J. Biol. Chem 2000 275 2247 2250 10644669 10.1074/jbc.275.4.2247

[115] Bronchal S Nain CK Prasad KK Nada R Sharma AK Sinha SK Singh K Functional and morphological alterations in small intestine mucosa of chronic alcoholics J. Gastroenterol. Hepatol 2008 23 e43 e48 17683494 10.1111/j.1440-1746.2007.05080.x

[116] Kravos M Malesic I Kinetics and isoforms of serum glutamate dehydrogenase in alcoholics Alcohol Alcohol 2008 43 281 286 18304992 10.1093/alcalc/agn010

[117] Lakshmi DS Anuradha CV Mitochondrial damage, cytotoxicity and apoptosis in iron-potentiated alcoholic liver fibrosis: Amelioration by taurine Amino Acids 2010 38 869 879 19381777 10.1007/s00726-009-0293-0

[118] Sastre J Serviddio G Pereda J Minana JB Arduini A Vendemiale G Poli G Pallardo FV Vina J Mitochondrial function in liver disease Front. Biosci 2007 12 1200 1209 17127373 10.2741/2138

[119] Hajnoczky G Buzas CJ Pacher P Hoek JB Rubin E Alcohol and mitochondria in cardiac apoptosis: Mechanisms and visualization Alcohol Clin. Exp. Res 2005 29 693 701 15897712 10.1097/01.alc.0000163493.45344.7a

[120] White AM What happened? Alcohol, memory blackouts, and the brain Alcohol Res. Health 2003 27 186 196 15303630 PMC6668891

[121] Davis KM Wu JY Role of glutamatergic and GABAergic systems in alcoholism J. Biomed. Sci 2001 8 7 19 11173971 10.1007/BF02255966

[122] Koch-Weser J Sellers EM Kalent HL Alcohol intoxication and withdrawal N. Engl. J. Med 1976 294 757 762 3733 10.1056/NEJM197604012941405

[123] Kosten TR O’Connor PG Management of drug and alcohol withdrawal N. Engl. J. Med 2003 348 1786 1795 12724485 10.1056/NEJMra020617

[124] Brust JCM Ethanol and cognition: Indirect effects, neurotoxicity and neuroprotection: A review Int. J. Environ. Res. Public Health 2010 7 1540 1557 20617045 10.3390/ijerph7041540 PMC2872345

[125] Obenier JA Bouldin TW Crews FT Binge ethanol exposure in adult rats causes necrotic cell death Alcohol Clin. Exp. Res 2002 26 547 557 11981132

[126] Silvers JM Tokunaga S Berry RB White AM Matthews DB Impairment in spatial learning and memory: Ethanol, allopregnanolone, and the hippocampus Brain Res. Brain Res. Rev 2003 43 275 284 14629930 10.1016/j.brainresrev.2003.09.002

[127] Grant I Alcohol and the brain: Neuropsychological correlates J. Consult. Clin. Psychol 1987 55 310 324 3597943 10.1037//0022-006x.55.3.310

[128] Israel Y Kalant H Effect of ethanol on the transport of sodium in frog skin Nature 1963 200 476 478 14076738 10.1038/200476a0

[129] Blum K Noble EP Sheridan PJ Montgomery A Ritchie T Jagadeesawaran P Nogami H Briggs AH Cohn JB Allelic association of human dopamine D2 and Gaba receptor gene in alcoholism JAMA 1990 263 2055 2060 1969501

[130] Kluge H Neumann J Seidel K Biochemical mechanisms for the effect of alcohol on the brain Psychiatr. Neurol. Med. Psychol (Leipz) 1979 31 65 73 36641

[131] Kharchenko NK Dopamine content in blood and activity of alcohol-transforming enzymes in alcoholism Ukr. Biokhim. Zh 1997 69 87 92 9463245

[132] Smith AA Interaction of biogenic amines with ethanol Adv. Exp. Med. Biol 1975 56 265 275 238368 10.1007/978-1-4684-7529-6_13

[133] Weiner H Relationship between 3,4-dihydroxyphenylacetaldehyde levels and tetrahydropapaveroline formation Alcohol Clin. Exp. Res 1978 2 127 131 350075 10.1111/j.1530-0277.1978.tb04712.x

[134] Haorah J Ramírez SH Floreani N Gorantla S Morsey B Persidsky Y Mechanism of alcohol-induced stress and neuronal injury Free Radic. Biol. Med 2008 45 1542 1550 18845238 10.1016/j.freeradbiomed.2008.08.030 PMC2605399

[135] Mansouri A Demeilliers C Amsellen S Pessayre D Fromenty B Acute ethanol administration oxidatively damages and depletes mitochondrial DNA in mouse liver, brain, heart, and skeletal muscles: Protective effects of antioxidants J. Pharmacol. Exp. Ther 2001 298 737 743 11454938

[136] Jung ME Agarwal R Simpkins JW Ethanol withdrawal posttranslationally decreases the activity of cytochrome *c* oxidase in an estrogen reversible manner Neurosci. Lett 2007 41 160 164 17320290 10.1016/j.neulet.2007.01.065 PMC2081971

[137] Jung ME Simpkins JW Wilson AM Downey HF Mallet RT Intermittent hypoxia conditioning prevents behavioral deficit and brain oxidative stress in ethanol-withdrawn rats J. Appl. Physiol 2008 105 510 517 18499779 10.1152/japplphysiol.90317.2008 PMC2519950

[138] Lee do Y Lee KS Lee HJ Jung HY Lee JY Lee SH Youn YC Seo KM Lee JH Lee WB Kim SS Alcohol enhances Abeta42-induced neuronal cell death through mitochondrial dysfunction FEBS Lett 2008 582 4185 4190 19026642 10.1016/j.febslet.2008.11.007

[139] Streissguth AP Landersman-Dwyer S Martin JC Smith DW Teratogenic effects of alcohol in humans and laboratory animals Science 1980 209 353 361 6992275 10.1126/science.6992275

[140] Clarren SK Alvord EC Sumi SM Streissguth AP Smith DW Brain malformations related to prenatal exposure to ethanol J. Pediatr 1978 92 64 67 619080 10.1016/s0022-3476(78)80072-9

[141] Riikonen RS Nokelainen P Valkonen K Kolehmainen AI Kumpulainen KI Könönen M Vanninen RL Kuikka JT Deep serotonergic and dopaminergic structures in fetal alcoholic syndrome: A study with nor-beta-CIT-single-photon emission computed tomography and magnetic resonance imaging volumetry Biol. Psychiatry 2005 57 1565 1572 15953494 10.1016/j.biopsych.2005.01.029

[142] Druse MJ Tajuddin NF Gillespie RA Le P The effects of ethanol and the serotonin(1A) agonist ipsapirone on the expression of the serotonin(1A) receptor and several antiapoptotic proteins in fetal rhombencephalic neurons Brain Res 2006 1092 79 86 16687129 10.1016/j.brainres.2006.02.065

[143] Sari Y Powrozek T Zhou FC Alcohol deters the outgrowth of serotonergic neurons at midgestation J. Biomed. Sci 2001 8 119 125 11173985 10.1007/BF02255980

[144] Mayordomo F Renau-Piqueras J Megias L Guerri C Iborra FJ Azorin I Ledig M Cytochemical and stereological analysis of rat cortical astrocytes during development in primary culture. Effect of prenatal exposure to ethanol Int. J. Dev. Biol 1992 36 311 321 1326314

[145] Devi BG Henderson GI Frosto TA Schenker S Effect of ethanol on rat fetal hepatocytes: Studies on cell replication, lipid peroxidation and glutathione Hepatology 1993 18 648 659 8359806

[146] Sanchis R Guerri C Alcohol-metabolizing enzymes in placenta and fetal liver: Effect of chronic ethanol intake Alcohol Clin. Exp. Res 1986 10 39 44 3515990 10.1111/j.1530-0277.1986.tb05611.x

[147] Maheshwari RK Singh AK Gaddipati J Srimal RC Multiple biological activities of curcumin: A short review Life Sci 2006 78 2081 2087 16413584 10.1016/j.lfs.2005.12.007

[148] Priyadarsini KI Maity DK Naik GH Kumar MS Unnikrishnan MK Satav JG Mohan H Role of phenolic O-H and methylene hydrogen on the free radical reactions and antioxidant activity of curcumin Free Radic. Biol. Med 2003 35 475 484 12927597 10.1016/s0891-5849(03)00325-3

[149] Churchill EN Disatnik MH Mochly-Rosen D Time-dependent and ethanol-induced cardiac protection from ischemia mediated by mitochondrial translocation of varepsilon PKC and activation of aldehyde dehydrogenase2 J. Mol. Cell Cardiol 2009 46 278 284 18983847 10.1016/j.yjmcc.2008.09.713 PMC2675554

[150] Cannon CP Braunwald E McCabe CH Rader DJ Rouleau JL Belder R Joyal SV Hill KA Skene AM Pravastatin or atorvastatin evaluation and infection therapy-thrombolysis in myocardial infarction 22 investigators N. Engl. J. Med 2004 350 1495 1504 15007110 10.1056/NEJMoa040583

[151] Blake GJ Ridker PM Novel clinical markers of vascular wall inflammation Circulation Res 2001 89 763 771 11679405 10.1161/hh2101.099270

[152] Clarke R Daly L Robinson K Naughten E Cahalane S Fowler B Graham I Hyperhomocysteinemia: An independent risk factor for vascular disease N. Engl. J. Med 1991 324 1149 1155 2011158 10.1056/NEJM199104253241701

[153] Brisdelli F D’Andrea G Bozzi A Resveratrol: A natural polyphenol with multiple chemopreventive properties Curr. Drug Metab 2009 10 530 46 19702538 10.2174/138920009789375423

[154] Cheng TH Liu JC Lin H Shih NL Chen YL Huang MT Chan P Cheng CF Chen JJ Inhibitory effect of resveratrol on angiotensin II-induced cardiomyocyte hypertrophy Naunyn Schmiedebergs Arch. Pharmacol 2004 369 239 244 14663554 10.1007/s00210-003-0849-6

[155] Chan AY Dolinsky VW Soltys CL Viollet B Baksh S Light PE Dyck JR Resveratrol inhibits cardiac hypertrophy *via* AMP-activated protein kinase and Akt J. Biol. Chem 2008 283 24194 24201 18562309 10.1074/jbc.M802869200 PMC3259789

[156] Serafini M Maiani G Ferro-Luzzi A Alcohol-free red wine enhances plasma antioxidant capacity in humans J. Nutr 1998 128 1003 1007 9614160 10.1093/jn/128.6.1003

[157] Opie LH Lecour S The red wine hypothesis: From concepts to protective signalling molecules Eur Heart J 2007 28 1683 1693 17561496 10.1093/eurheartj/ehm149

[158] Hung LM Chen JK Lee RS Liang HC Su MJ Beneficial effects of astringinin, a resveratrol analogue, on the ischemia and reperfusion damage in rat heart Free Radic. Biol. Med 2001 30 877 883 11295530 10.1016/s0891-5849(01)00474-9

[159] Fauconneau B Waffo-Teguo P Huguet F Barrier L Decendit A Merillon JM Comparative study of radical scavenger and antioxidant properties of phenolic compounds from Vitis vinifera cell cultures using *in vitro* tests Life Sci 1997 61 2103 2110 9395251 10.1016/s0024-3205(97)00883-7

[160] El-Assal O Hong F Kim W-H Radaeva S Gao B IL-6 deficient mice are susceptible to ethanol-induced hepatic steatosis: IL-6 protects against ethanol-induced oxidative stress and mitochondrial permeability transition in the liver Cell Mol. Immunol 2004 1 205 211 16219169

[161] Hong F Kim WH Tian Z Jaruga B Ishac E Shen X Gao B Elevated interleukin-6 during ethanol consumption acts as a potential endogenous protective cytokine against ethanol-induced apoptosis in the liver: Involvement of induction of Bcl-2 and Bcl-x(L) proteins Oncogene 2002 21 32 43 11791174 10.1038/sj.onc.1205016

[162] Lowe ED Gao GY Johnson LN Keung WM Structure of didzin, a naturally occurring anti-alcohol-addiction agent, in complex with human mitochondrial aldehyde dehydrogenase J. Med. Chem 2008 51 4482 4487 18613661 10.1021/jm800488j

[163] Kim YC Jung YS Kim SK Effect of betaine supplementation on changes in hepatic metabolism of sulfur-containing amino acids and experimental cholestasis induced by alphanaphthylisothiocyanate Food Chem. Toxicol 2005 43 663 670 15778005 10.1016/j.fct.2004.12.015

[164] Craig SA Betaine in human nutrition Am. J. Clin. Nutr 2004 80 539 549 15321791 10.1093/ajcn/80.3.539

[165] Zhang P Gong ZJ Wang LW Sun XM Zhou XR Effects of Betaine on hyperhomocysteinemia and lipid peroxidation in rats with ethanol-induced liver injury Zhongxiyi Jiehe Ganbing Zazhi 2006 16 30 32

[166] Ji C Shinohara M Vance D Than TA Ookhtens M Chan C Kaplowitz N Effect of transgenic extrahepatic expression of betaine-homocysteine methyltransferase on alcohol or homocysteine-induced fatty liver Alcohol Clin. Exp. Res 2008 32 1049 1058 18498552 10.1111/j.1530-0277.2008.00666.x PMC2596885

[167] Cantoni GL Activation of methionine for transmethylation J. Biol. Chem 1951 189 745 754 14832292

[168] Mato JM Alvarez L Ortiz P Pajares MA S-adenosylmethionine synthesis: Molecular mechanisms and clinical implications Pharmacol. Ther 1997 73 265 280 9175157 10.1016/s0163-7258(96)00197-0

[169] Santamaria E Avila MA Latasa MU Rubio A Martin-Duce A Lu SC Mato JM Corrales FJ Functional proteomics of nonalcoholic steatohepatitis: Mitochondrial proteins as targets of S-adenosylmethionine Proc. Natl. Acad. Sci. USA 2003 100 3065 3070 12631701 10.1073/pnas.0536625100 PMC152247

[170] Bailey SM Robinson G Pinner A Chamlee L Ulasova E Pompilius M Page GP Chhieng D Jhala N Landar A Kharbanda KK Ballinger S Darley-Usmar V S-adenosylmethionine prevents chronic alcohol-induced mitochondrial dysfunction in the rat liver Am. J. Gastrointest. Liver Physiol 2006 291 G857 G867 10.1152/ajpgi.00044.2006 16825707

